# Seasonal dynamics and molecular differentiation of three natural *Anopheles* species (Diptera: Culicidae) of the Maculatus group (Neocellia series) in malaria hotspot villages of Thailand

**DOI:** 10.1186/s13071-020-04452-0

**Published:** 2020-11-11

**Authors:** Suchada Sumruayphol, Tanawat Chaiphongpachara, Yudthana Samung, Jiraporn Ruangsittichai, Liwang Cui, Daibin Zhong, Jetsumon Sattabongkot, Patchara Sriwichai

**Affiliations:** 1 Department of Medical Entomology, Faculty of Tropical Medicine, Mahidol University, Bangkok, Thailand; 2 Division of Infectious Diseases, Department of Internal Medicine, Morsani College of Medicine, University of South Florida, Tampa, FL 33612 USA; 3 Program in Public Health, University of California at Irvine, Irvine, CA 92697 USA; 4 Mahidol Vivax Research Unit, Faculty of Tropical Medicine, Mahidol University, Bangkok, Thailand

**Keywords:** *Anopheles maculatus*, *Anopheles pseudowillmori*, *Anopheles sawadwongporni*, Seasonal dynamic, Cytochrome *c* oxidase subunit 1, Internal transcribed spacer 2, Species complex

## Abstract

**Background:**

*Anopheles sawadwongporni* Rattanarithikul & Green, *Anopheles maculatus* Theobald and *Anopheles pseudowillmori* (Theobald) of the *Anopheles maculatus* group (Diptera: Culicidae) are recognized as potential malaria vectors in many countries from the Indian subcontinent through Southeast Asia to Taiwan. A number of malaria vectors in malaria hotspot areas along the Thai-Myanmar border belong to this complex. However, the species distribution and dynamic trends remain understudied in this malaria endemic region.

**Methods:**

Mosquitoes of the Maculatus group were collected using CDC light traps every other week from four villages in Tha Song Yang District, Tak Province, Thailand from January to December 2015. Adult female mosquitoes were morphologically identified on site using taxonomic keys. Molecular species identification was performed by multiplex PCR based on the internal transcribed spacer 2 (ITS2) region of ribosomal DNA (rDNA) and sequencing of the *cox*1 gene at a DNA barcoding region in a subset of 29 specimens.

**Results:**

A total of 1328 *An. maculatus* (*sensu lato*) female mosquitoes were captured with *An. maculatus*, *An. sawadwongporni* and *An. pseudowilmori* accounting for 75.2, 22.1 and 2.7% respectively. The field captured mosquitoes of the Maculatus group were most abundant in the wet season and had a preferred distribution in villages at higher elevations. The phylogenetic relationships of 29 *cox*1 sequences showed a clear-cut separation of the three member species of the Maculatus group, with the *An. pseudowillmori* cluster being separated from *An. sawadwongporni* and *An. maculatus*.

**Conclusions:**

This study provides updated information for the species composition, seasonal dynamics and microgeographical distribution of the Maculatus group in malaria-endemic areas of western Thailand. This information can be used to guide the planning and implementation of mosquito control measures in the pursuance of malaria transmission.
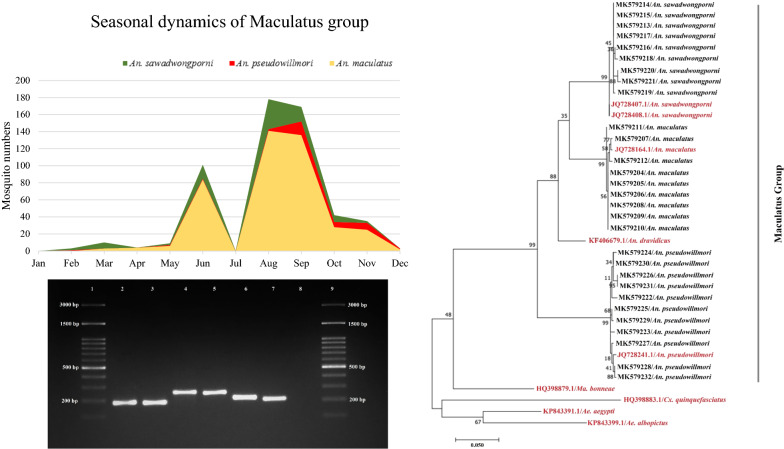

## Background

Malaria is a serious parasitic disease caused by the protozoan parasites of the genus *Plasmodium* that occurs mainly throughout tropical and subtropical zones [1]. In 2018, the World Health Organization estimated that there were 228 million cases of malaria, down from 231 million cases in 2017, and 405,000 deaths from malaria worldwide [2]. *Anopheles* mosquitoes (Diptera: Culicidae) are the sole vectors of malaria. Globally, a total of 494 species of the subfamily Anophelinae are currently recognized, which are divided into the following eight subgenera: *Anopheles* (190 species), *Baimaia* (1 species), *Cellia* (225 species), *Christya* (2 species), *Kerteszia* (12 species), *Lophopodomyia* (6 species), *Nyssorhynchus* (40 species), and *Stethomyia* (5 species) [3]. Approximately 70 species of formally recognized *Anopheles* are human malaria vectors and 40 species are dominant malaria vectors [4, 5].

Different species of *Anopheles* mosquitoes play different roles in malaria transmission in each area. Each *Anopheles* species has particular characteristics in biology, ecology, and behavior such as host preference, biting indoors or outdoors (endophagic/exophagic), resting behavior (exophilic/endophilic), longevity, and larval habitat preference [6]. Therefore, the first key for the control of malaria vectors is the knowledge of the correct vector species that can transmit malaria. However, species identification of *Anopheles* using standard morphological methods is difficult in those with similar morphologies including the various species complexes in Southeast Asia [7]. Additionally, the collection of adult mosquitoes in the field using traps often causes damage to the specimens, resulting misidentification [8].

The Maculatus group as a large species assemblage consists of nine member species including *Anopheles dispar* Rattanarithikul & Harbach, *Anopheles greeni* Rattanarithikul & Harbach, *Anopheles pseudowillmori* (Theobald), and *Anopheles willmori* (James), and five species assigned to two subgroups, the Maculatus subgroup which consists of *Anopheles dravidicus* Christophers and *Anopheles maculatus* Theobald, and the Sawadwongporni subgroup which consists of *Anopheles notanandai* Rattanarithikul & Green, *Anopheles rampae* Harbach & Somboon, and *An. sawadwongporni* Rattanarithikul & Green [3, 9]. The member species in this group are distributed throughout many countries from the Indian subcontinent through Southeast Asia to Taiwan and are recognized as being associated with malaria [7, 10, 11]. However, microgeographical distribution and seasonal dynamics of this species complex are unclear, which are an obstacle for their control. Since each member species has similar morphological characteristics, species identification using morphological methods is difficult [7].

In Thailand, seven member species of the Maculatus group have been reported, of which three (*An. sawadwongporni*, *An. maculatus* and *An. pseudowillmori*) are the potential malaria vectors [12–14]*.* In malaria transmission hotspot areas of Thailand such as Tak Province, these species are found in sympatry [12–15]. Although all these three *Anopheles* species have similar morphological characteristics, *An. maculatus* and *An. sawadwongporni* can be distinguished by a tuft of black scales on the wing vein bifurcation of radius *2* and radius *3*, while *An. pseudowillmori* can be identified by the pattern of scales on the abdomen and wings [9]. Although species identification during routine entomological surveillance can be performed down to the subgenus level, resolution of the species complex requires in-depth morphological analysis by an expert entomologist. Damage of the wing scales, especially the characters needed for species identification, often occurs during sample collection in the rainy season. Thus, molecular techniques are a powerful tool for the identification of morphologically similar species or morphologically indistinguishable species, and have become increasingly popular [7, 16, 17]. Such methods include PCR, which is relatively quick, straightforward, accurate, and reliable [18]. Sequence markers that have been used in previous research for the identification of mosquito species include the internal transcribed spacer 2 (ITS2) of ribosomal DNA (rDNA) [16, 18, 19], the second and third domains (D2 and D3) of the rDNA *28S* gene [16], and cytochrome *c* oxidase subunits 1 and 2 (*cox*1 and *cox*2) of the mitochondrial DNA [7, 20]. The *cox*1 gene is considered one of the most effective molecular markers because of frequent base substitutions in the third codon position, a lack of introns, limited exposure to recombination, and a haploid mode of inheritance. It is therefore commonly used in barcoding studies for the species identification of mosquito vectors [21, 22]. In Thailand, *cox*1 was used for DNA barcoding identification of sibling species of *Anopheles* mosquitoes including the Barbirostris group [22], the Hyrcanus group [23], and the Leucosphyrus group [24]. However, the identification of *Anopheles* species using the *cox*1 gene alone is not sufficient to make precise conclusions, and thus should be combined with alternative markers to increase the accuracy. Walton et al. [18] developed an effective PCR-based identification method to distinguish five member species of the Maculatus group in Thailand.

The present study aims to provide updated information on the natural distribution of the *An. maculatus* group on the village scale and assess the taxonomic status of naturally captured species of the Maculatus group in a malaria endemic area in Tak Province, Thailand using two molecular approaches: multiplex PCR of ITS2 and sequencing of the DNA barcode region in the *cox*1 gene. The sample sources were also used to define the vector seasonal dynamics and the composition of the Maculatus complex. The results are consistent with the distribution of the Maculatus group in high-transmission area implicating their contribution to malaria transmission.

## Methods

### Mosquito collection and morphological identification

**Fig. 1:**
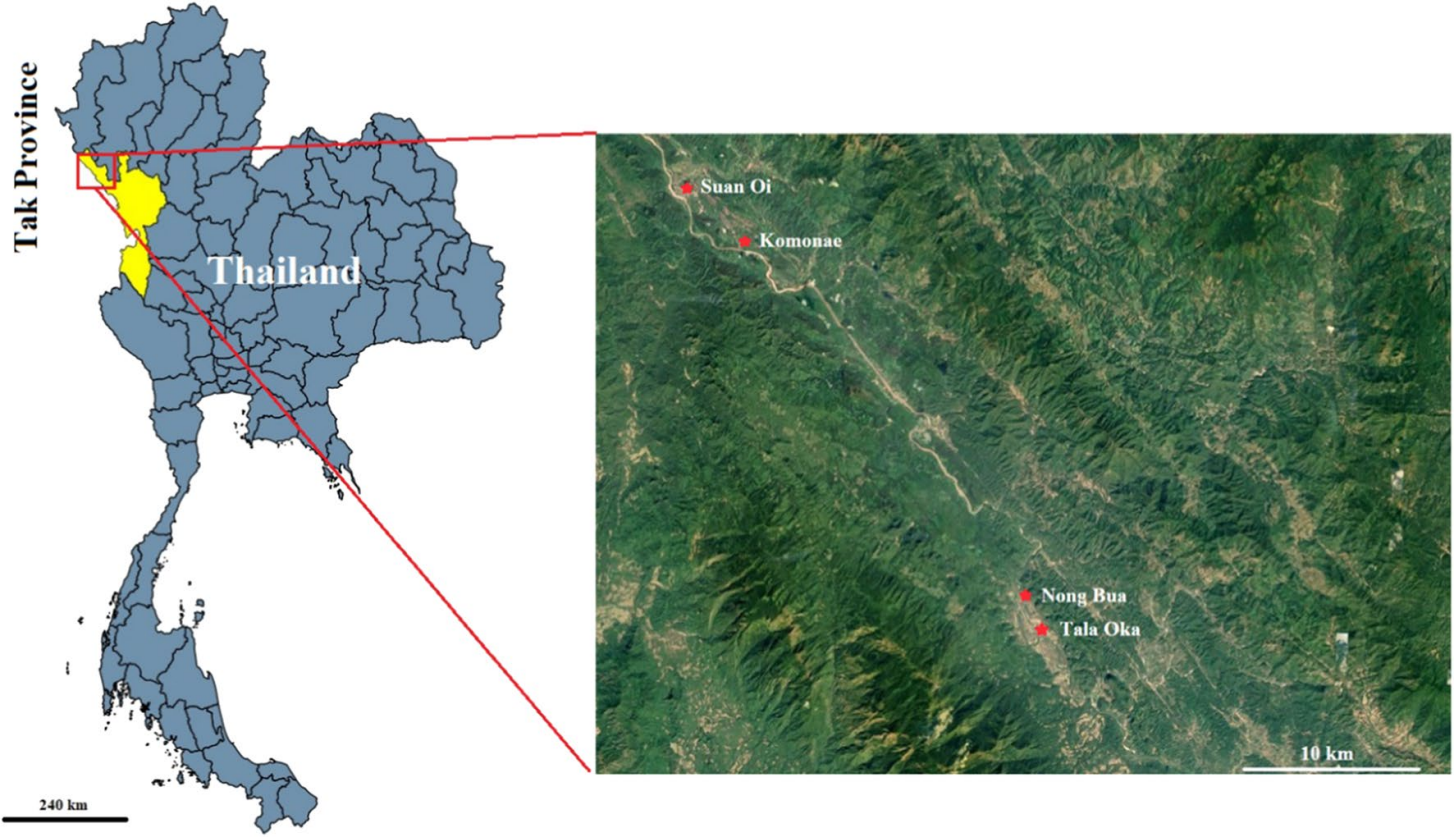
Map showing locations of the four villages along the Thai-Myanmar border (Suan Oi, Komonae, Nong Bua and Tala Oka) where mosquitoes were collected

Mosquitoes were collected using CDC light traps with a 6-volt battery (BioQuip, Rancho Dominguez, CA, USA) with 1 kg of dry ice from four villages, Suan Oi (SO) (17°33ʹ36.5ʺN, 97°55ʹ12.5ʺE), Komonae (KN) (17°31ʹ57.4ʺN, 97°56ʹ57.9ʺE), Nong Bua (NB) (17°20ʹ24.8ʺN, 98°06ʹ24.6ʺE), and Tala Oka (TO) (17°19ʹ24.5ʺN, 98°06ʹ58.0ʺE), in the Tha Song Yang District, Tak Province, Thailand (Fig. 1) between January and December 2015. The traps were set in the selected high-transmission villages KN and SO and low-transmission villages TO and NB. All mosquitoes were captured between 18:00 h and 6:00 h in three consecutive nights for every other week by hanging the traps approximately 1.5 m above the ground and 20 m away from houses. In total, 30 replacements/week/village (15 indoor and 15 outdoor) from 30 houses in each village with reported cases of malaria were selected for mosquito trapping. Bottle programmable rotator CDC light traps (BioQuip) were set to collect the mosquitoes every 2 h for 6 replacements/week/village. They were placed outdoors from additional 24 selected houses in each village. In the morning, mosquito samples were transferred from the CDC light traps into 60 ml specimen containers, kept in dry ice boxes, and sent to the Department of Medical Entomology, Faculty of Tropical Medicine, Mahidol University, Bangkok for species identification. Sources of samples were recorded with details of the trap types, location, gravidity, and geographical information at each village. Adult females of the maculatus group were morphologically identified on site using taxonomic keys [9, 25]. Maculatus group samples were focused and reconfirmed in the laboratory by morphology. Samples were then preserved dry at – 20 °C for molecular identification. The meteorological parameters of temperature, humidity, and rainfall were obtained from the local climatology division (code station 376202), Meteorological Department, Ministry of Information and Communication Technology, Bangkok, Thailand. The station is localized in Mae Sod District, Tak Province, about 60 km away from the study site.

### Malaria parasite detection in the mosquitoes

Collected *Anopheles* mosquitoes were kept at – 20 °C until the detection of malaria parasite sporozoites by using an enzyme-linked immunosorbent assay (ELISA). Field-captured Maculatus group mosquitoes were examined for circumsporozoite (CS) proteins of *Plasmodium falciparum, P. vivax*-210 (PV210), and *P. vivax*-247 (PV247) [26].

### DNA extraction and polymerase chain reaction (PCR)

For a subset of 29 *Anopheles* specimens that were confirmed by expert entomologists using morphological characteristics, including nine *An. sawadwongporni* individuals (SAW1–9), nine *An. maculatus* individuals (MAC1–9), and 11 *An. pseudowillmori* individuals (PSE1–11), genomic DNA was extracted using the PureLink® Genomic DNA Mini Kit (Invitrogen, Carlsbad, USA) according to the manufacturer’s protocol. DNA was stored in separate 1.5 ml microcentrifuge tubes at – 20 °C until further analysis. Information on the 29 mosquito specimens is provided in Additional file 1: Table S1.

The *cox*1 gene (658 bp fragment) was amplified using the forward primer *LepF1* (5ʹ-ATT CAA CCA ATC ATA AAG ATA TTG G-3ʹ) and reverse primer *LepR1* (5ʹ-TAA ACT TCT GGA TGT CCA AAA AAT CA-3ʹ) [27]. The ITS2 region was amplified using four primers including the universal forward 5.8F (5ʹ-ATC ACT CGG CTC GTG GAT CG-3ʹ), SAW for *An. sawadwongporni* (5ʹ-ACG GTC CCG CAT CAG GTG C-3ʹ), MAC for *An. maculatus* (5ʹ-GAC GGT CAG TCT GGT AAA GT-3ʹ), and PSEU for *An. pseudowillmori* (5ʹ-GCC CCC GGG TGT CAA ACA G-3 ʹ) [18]. For the *cox*1 gene, PCR reactions contained 5 µl 10× PCR buffer, 50 µM dNTPs, 2.5 mM MgCl_2_, 1.2 U Platinum® *Taq* DNA polymerase (Invitrogen), 0.2 µM of each primer, 5 µl of DNA template, and the remaining of ultrapure H_2_O. Initial denaturation was performed at 94 °C for 1 min followed by five cycles at 94 °C for 30 s, 45 °C for 40 s, and 72 °C for 1 min, then amplification was performed using 35 cycles of 95 °C for 30 s, 55 °C for 40 s, and 72 °C for 1 min, with a final extension at 72 °C for 10 min. For the ITS2 region, PCR reactions contained 2.5 µl 10× PCR buffer, 200 µM dNTPs, 2.5 mM MgCl_2_, 2 U Platinum® *Taq* DNA polymerase (Invitrogen), 0.2 mM of each primer, 2 µl of DNA template, and the remaining volume of ultrapure H_2_O. PCR cycling conditions for ITS2 were described by Walton et al. [18] as follows: an initial denaturation step at 94 °C for 5 min; then 35 cycles of 94 °C for 1 min, 61 °C for 30 s, and 72 °C for 30 s; followed by a final extension at 72 °C for 5 min. PCR products of all specimens were electrophoresed in 1.5% agarose gels stained with Novel Juice supplied in 6× DNA Loading Buffer (BIO-HELIX Co., Keelung, Taiwan) and photographed with the Gel Doc™ XR+ system (Bio-Rad, Hercules, USA), to verify band sizes. All *cox*1 PCR products showing positive clear bands were sent for sequencing to Macrogen Korea Inc. (Seoul, Rep. of Korea). For accuracy, sequencing was done for both strands. ITS2 PCR products were identified by comparison with the OneMARK 100 DNA ladder (100–3000 bp; BIO-HELIX Co.). A specific band was expected for each species (242 bp for *An. sawadwongporni*, 180 bp for *An. maculatus*, and 203 bp for *An. pseudowillmori*) [18].

### Sequence alignment and phylogenetic analyses

Each *cox*1 sequence was compared with previously published sequences in GenBank using the standard nucleotide Basic Logical Alignment Search Tool, available at https://blast.ncbi.nlm.nih.gov/Blast.cgi and in The Barcode of Life Database (BOLD) available at https://www.barcodinglife.org. The chromatograms (traces) of *cox*1 sequences were visualized and manually edited using Chromas software version 2.6.6 (https://technelysium.com.au/wp/). Multiple *cox*1 sequence alignment was performed using Clustal X version 2.1 [28]. Intraspecific and interspecific pairwise sequence divergences of all individuals were calculated using the distance model Kimura 2-parameter (K2P), available within the Molecular Evolutionary Genetics Analysis (MEGA) software version 7 based on the number of nucleotide substitutions per site between two DNA sequences [29]. The detailed sequences of 29 individuals of three members of the Maculatus group were submitted and uploaded to GenBank (Additional file 2: Table S2). A phylogenetic tree was built to provide a graphical representation of the clustering pattern among member species of the Maculatus group. To identify the genetic relationships among the Maculatus group by phylogenetic analysis, we used the reference genome for other member species in the Maculatus group from GenBank (Additional file 2: Table S2). The sequences of *Aedes aegypti*, *Ae. albopictus*, *Culex quinquefasciatus*, and *Mansonia bonneae* were used as outgroup species (Additional file 2: Table S2) . Phylogenetic analysis was performed by using the maximum likelihood method with 1000 bootstrap replications implemented in the MEGA 7.0 software. The general time reversible plus gamma distributed with invariant sites (GTR + G + I) was used as the nucleotide substitution model.

## Results

### Morphological identification of *An. maculatus* group mosquitoes

**Fig. 2:**
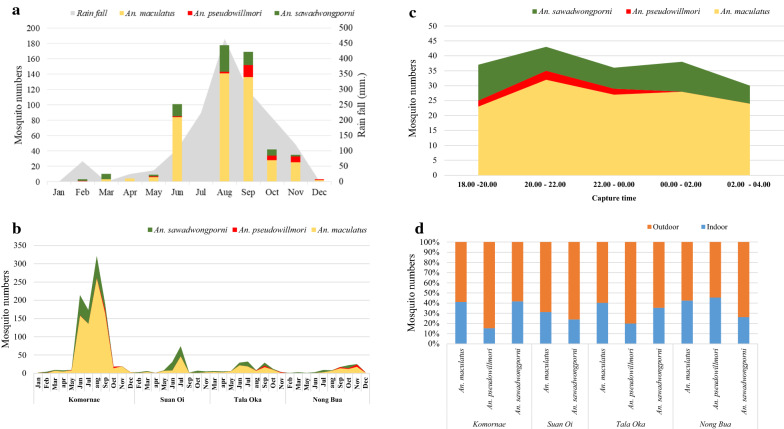
*Anopheles maculatus* group captured from the four villages in 2015 identified by morphology. **a** Monthly collection patterns of *An. maculatus* group with the mean of rainfall (mm). **b** Mosquito numbers collected from individual villages. **c** Time collection of *An. maculatus* group captured by the outdoor CDC-light timing rotator trap. **d** Indoor and outdoor proportions of *An. maculatus* group from the four villages

In 2015, a total of 7519 *Anopheles* mosquitoes were collected using the two different light traps. Twenty-four species were identified morphologically, among which *An. minimus*, *An. culicifacies, An. maculatus*, *An. annularis* were the most abundant, accounting for 49.5, 14.0, 13.3 and 11.3% of the total collection, respectively (Table 1). In total, 1328 *An. maculatus (s.l.)* were captured from the four villages. Two species of the Maculatus group, *An. maculatus* and *An. sawadwongporni*, were initially assigned on site by the field sample collection team using morphological characters. However, morphological reconfirmation by expert entomologists in the medical entomology laboratory revealed that 75.2, 22.1 and 2.7% of these specimens were *An. maculatus*, *An. sawadwongporni* and *An. pseudowilmori*, respectively (Table 2). The monthly dynamics of the Maculatus group captured from the four villages showed two peaks during the rainy season with the main peak occurring in August to September (Fig. 2a). While most samples of this species group were collected from the high-transmission sites (KN and SO), *An. maculatus* was the major species captured from both high- and low-transmission villages. KN village had the highest abundance of the Maculatus group with the highest proportion of *An. maculatus* 58.6% (778/1328), which peaked in August. In other villages, the captured *An. maculatus* ranged from 4.4% to 6.7%. *Anopheles sawadwongporni* had the same seasonal trend with high prevalence during June-August. The majority (13.10%, 174/1328) of the *An. sawadwongporni* samples was collected in KN, whereas 1.7–13.1% were collected in other villages. *Anopheles pseudowilmori* was found in three of the four villages except SO, with the highest numbers of 17 in KN followed by 12 in NB (Fig. 2b). The rotating CDC light traps were used to monitor the times of mosquito activities. The results showed that the first peak of the Maculatus group occurred at 20.00–22.00 h and the second peak at 00.00–2.00 h (Fig. 2c). Of all of the Maculatus species captured, 48.9% *An. maculatus,* 15.3% *An. sawadwongporni,* and 2.1% *An. pseudowilmori*, respectively, were collected by the outdoor traps (Fig. 2d, Table 2). Of the 1328 Maculatus species collected, 25 (1.9%) were blood-fed. For *An. maculatus*, 2.3% (20/999) were blood-fed, and the ratio of blood-fed *An. maculatus* (8.6%) was the highest in NB (Table 2).

**Table 1:** *Anopheles* mosquito species composition collected in the four villages (Komonae (KN), Suan Oi (SO), Tala Oka (TO) and Nong Bua (NB)) of western Thailand

Species	KN	SO	TO	NB	Total	%
*An. minimus*	2464	586	494	181	3725	49.54
*An. culicifacies*	231	262	341	220	1054	14.02
*An. maculatus*	778	74	89	58	999	13.29
*An. annularis*	36	6	577	232	851	11.32
*An. sawadwongporni*	174	63	34	22	293	3.90
*An. barbirostris*	4	2	120	61	185	2.46
*An. paeditaeniatus*	9	3	62	28	102	1.36
*An. tessellatus*	21	2	64	10	97	1.29
*An. kochi*	2	3	19	17	41	0.55
*An. vagus*		1	30	7	38	0.51
*An. pseudowillmori*	17		7	12	36	0.48
*An. jemesi*	11	2	15	3	31	0.41
*An. nigerrimus*	6	5	7		18	0.24
*Anopheles* spp*.*	2	2	5	9	18	0.24
*An. pujutensis*	5		2	1	8	0.11
*An. indefinitus*	2		2	2	6	0.08
*An. philipinensis*		1	2	1	4	0.05
*An. varuna*	3				3	0.04
*An. subpictus*	1		2		3	0.04
*An. kawari*	1			1	2	0.03
*An. campestris*			2		2	0.03
*An. dirus*				1	1	0.01
*An. stephensi*			1		1	0.01
*An. pseudojemsii*				1	1	0.01
Grand total	3766	1012	1874	867	7519

**Table 2:** Sex and female abdominal stages of the maculatus group collected from the light traps (indoor and outdoor)

Species	KN		SO			TO		NB			Total	%
	Blood	Empty	Blood	Empty	Male	Blood	Empty	Blood	Empty	Male		
*An. maculatus*	15	763	2	70	2	1	88	5	53		999	75.2
Indoor	4	274	1	19		1	28	3	20		350	26.4
Outdoor	11	489	1	51	2		60	2	33		649	48.9
*An. sawadwongporni*	1	173	1	62			34		21	1	293	22.1
Indoor		61		13			11		5		90	6.8
Outdoor	1	112	1	49			23		16	1	203	15.3
*An. pseudowillmori*		17					7		12		36	2.7
Indoor		2					1		5		8	0.6
Outdoor		15					6		7		28	2.1
Grand total	16	953	3	132	2	1	129	5	86	1	1328	100

### *Plasmodium* infectivity in the Maculatus group mosquitoes

ELISA analysis of all mosquitoes of the Maculatus group revealed that only one *An. maculatus* mosquito captured from an indoor light trap in KN village in August was positive for *P. vivax* (PV210).

### Molecular identification based on the species-specific ITS2 region

**Fig. 3:**
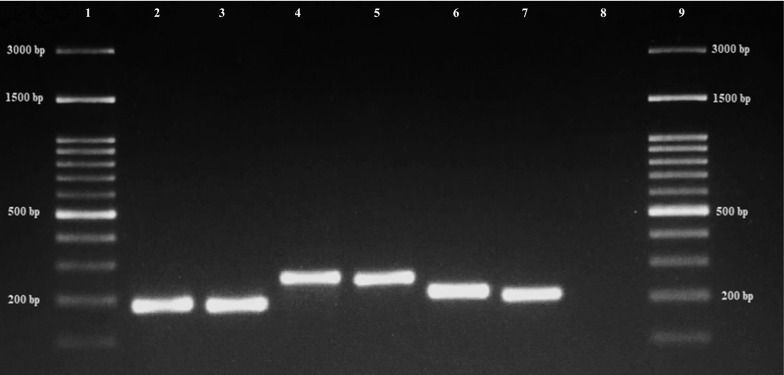
Identification of *An. sawadwongporni*, *An. maculatus* and *An. pseudowillmori* by PCR based on the ITS2 region. PCR products from three member species of the Maculatus group. Lanes 1 and 9: 100–3000 bp ladder; Lanes 2–3: *An. maculatus*; Lanes 4–5: *An. sawadwongporni*; Lanes 6–7: *An. pseudowillmori*; Lane 8: negative control

The 29 samples that were identified to the species level using morphological characteristics were investigated by multiplex PCR based on the ITS2 region. All *Anopheles* species in the Maculatus group were confirmed to match their morphological confirmation and *cox*1 sequence identification. The 9 *An. sawadwongporni* displayed the largest PCR band of 242 bp, followed by 11 *An. pseudowillmori* (203 bp), and 9 *An. maculatus* (180 bp) (Fig. 3).

### Mitochondrial *cox*1 gene sequence analysis

The *cox*1 sequences of the three species in the Maculatus group (658 bp) were analyzed between and within species. No noise or double peaks were found in the chromatograms of all obtained sequences. The adenosine and thymine (AT) content was similar for the three species: 69% in *An. sawadwongporni*; 68.5% in *An. maculatus*; and 67.8% in *An. pseudowillmori*. These sequences had the highest average A + T content at the first codon position, including 93.6% in *An. sawadwongporni*, 91.1% in *An. maculatus*, and 90.5% in *An. pseudowillmori*. The average intraspecific divergence of *An. sawadwongporni*, *An. maculatus*, and *An. pseudowillmori* based on K2P distances was 0.002, 0.004, and 0.008, respectively (Table 3), and the overall divergence among the three species was 0.069. Average interspecific divergences were 0.069 between *An. sawadwongporni* and *An. maculatus*, 0.114 between *An. sawadwongporni* and *An. pseudowillmori*, and 0.104 between *An. maculatus* and *An. pseudowillmori* (Table 3).

**Table 3:** Average of inter- and intraspecific pairwise divergence (K**2**P model) in three member species of the Maculatus group based on *cox*1 genes

Species	Average sequence divergence (minimum–maximum)		
	*An. sawadwongporni*	*An. maculatus*	*An. pseudowillmori*
*An. sawadwongporni*	0.004 (0.000–0.009)		
*An. maculatus*	0.069 (0.064–0.077)	0.002 (0.000–0.006)	
*An. pseudowillmori*	0.114 (0.108–0.126)	0.104 (0.099–0.111)	0.008 (0.000–0.012)

For species identification, sequences from the three members in the Maculatus group were compared with available sequences in the GenBank and BOLD systems. The 29 *cox*1 sequences generated from the three species of morphologically identified specimens showed more than 99% homology to *An. maculatus* (GenBank: JQ728164), *An. sawadwongporni* (GenBank: Q728408), and *An. pseudowillmori* (GenBank: JQ728241), respectively.

### Phylogenetic analysis

**Fig. 4:**
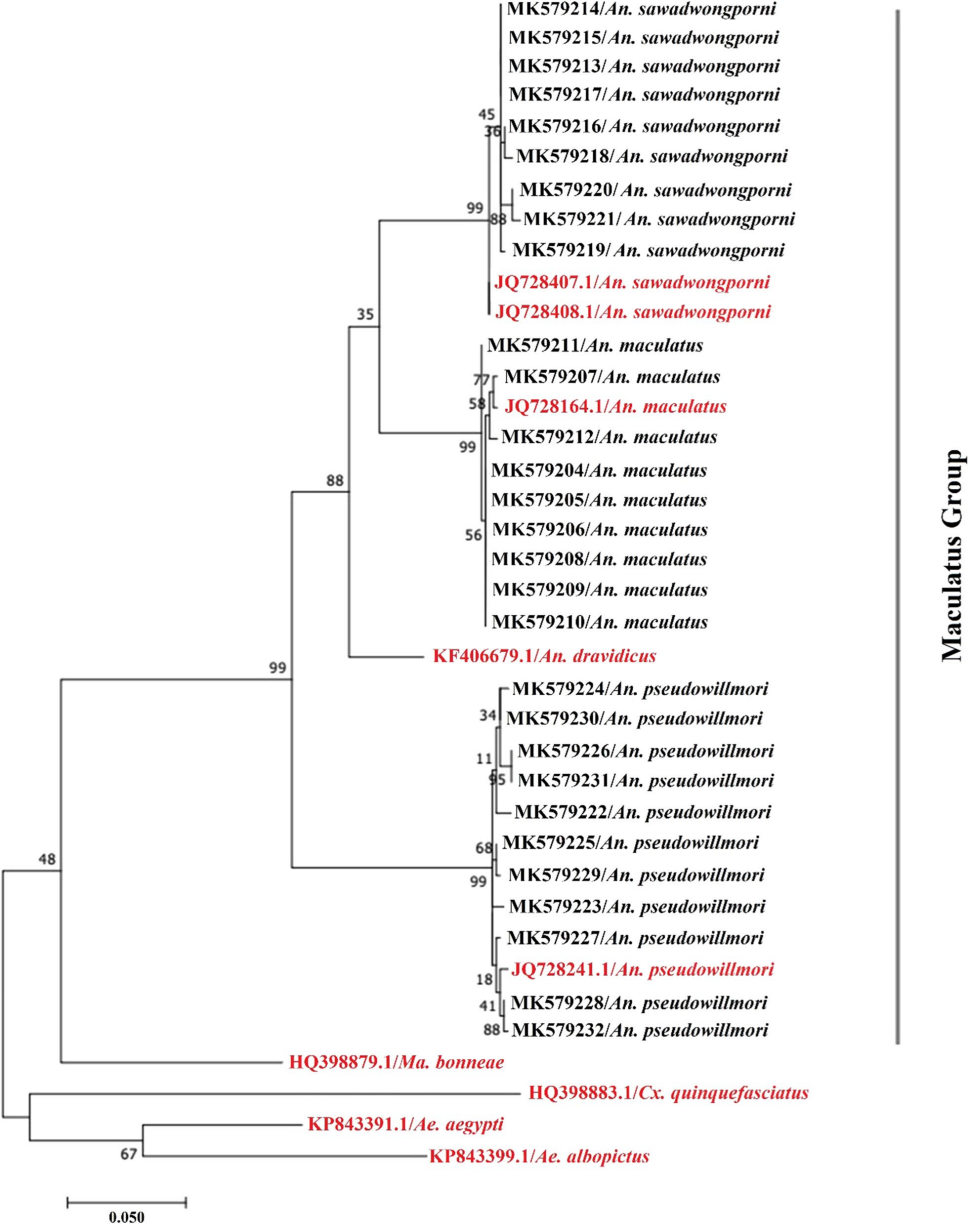
Phylogenetic tree based on maximum likelihood method with 1000 bootstrap replicates for *cox*1 barcode sequences of three species of Maculatus group in Thailand (*n* = 29, black labels) and of member species from China, India and Pakistan retrieved from GenBank (*n* = 6, red labels). The outgroup taxa include *Ae. aegypti*, *Ae. albopictus*, *Cx. quinquefasciatus* and *Ma. bonneae*

The relationships among the 29 *cox*1 sequences of *An. sawadwongporni*, *An. maculatus* and *An. pseudowillmori* of the Maculatus group in Thailand and 10 *cox*1 sequences retrieved from the GenBank database were analyzed by phylogeny (Fig. 4). The maximum likelihood tree showed a clear-cut separation between species members of the Maculatus group clusters. All specimens of the same species were grouped in individual clusters. The addition of a *cox*1 sequence of *An. dravidicus* from GenBank showed that it also belongs to the Maculatus group. The *An. pseudowillmori* cluster was separated from the *An. dravidicus* cluster and *An. sawadwongporni* and *An. maculatus* sub-cluster. Additionally, outgroup species including *Ae. aegypti*, *Ae. albopictus*, *Cx. quinquefasciatus*, and *Ma. bonneae* were clearly separated from clusters of *Anopheles* species in the Maculatus group. Altogether, the results of *cox*1 sequence analysis including the genetic divergence of K2P distances and phylogenetic tree clearly showed consistency with species identification derived from the species-specific ITS2 and morphological approach from the re-examination by entomological experts.

## Discussion

*Anopheles maculatus*, *An. sawadwongporni* and *An. pseudowillmori* are recognized as potential malaria vectors in many regions of the world including Thailand [13, 30], Motuo County in China’s Tibet [31], and southwestern China [32]. This study reported the dynamics of the Maculatus group in western Thailand, which is consistent with their malaria vector status with peak abundance in the wet season [13]. Herein, the study confirmed that there are at least three members of the Maculatus group with *An. maculatus* being the predominant potential malaria vector in this region. Although the same members of the Maculatus group were found in the neighboring Kayin State, Myanmar, *An. swadwongporni* was the main species there, suggesting species abundance within a small geographical region may vary [33]. This study further revealed that even within a small geographical area such as the four villages, vector abundance can vary drastically. More than 58% of the mosquito collection of the Maculatus group was from the village KN, which is near the forest and surrounded by mountains. The identification of a sporozoite-positive *An. maculatus* from KN also supports active malaria transmission in this village. Since mosquito control efforts consisting of indoor sprays of insecticides twice a year were conducted similarly in these villages by the local Vector-Borne Disease Control Unit, the differences in vector abundance may be due to the variations in local environment. With this information, more intensive mosquito control efforts should be undertaken in the village KN.

This study identified that the Maculatus group are generally more exophilic, though there were slight variations in the endophilic patterns of the different species within this group (Fig. 2d) as confirmed in another site of the Myanmar-Thai border [33]. Although the endo- or exophilicity is referred from the sites where they are collected (indoor *vs* outdoor) and may not reflect the true preference, this information nonetheless should be useful to guide the implementation of outdoor control efforts to prevent outdoor malaria transmission. Another important finding is the relatively persistent activity of the Maculatus group throughout the collection time in the night, though two peak collection times were noticed at 20.00–22.00 h and 00.00–2.00 h. The first peak time coincides with the family time before bed, suggesting that LLINs may offer ineffective protection for these early-biting mosquitoes. Alternative measures preventing mosquito biting before bedtime are needed [34].

Discriminating *Anopheles* species within species complexes can be difficult because of morphological similarity. Molecular techniques have been used to overcome this difficulty. This includes amplification of the ITS2 region of rDNA [18], D3 of rDNA, and *cox*2 of mitochondrial DNA [16]. *cox*1 could be used for DNA barcoding in mosquito identification [17], but has not been evaluated properly among member species of the Maculatus group. The present study determined the efficiency of using *cox*1 barcodes for the identification of *An. sawadwongporni*, *An. maculatus*, and *An. pseudowillmori* in Thailand, and established a group of sequences associated with the identified species. Species-specific multiplex PCR based on the ITS2 region was also used to confirm the findings based on *cox*1 analysis.

Phylogenetic relationships based on *cox*1 sequences clearly revealed grouping between species in the Maculatus group. The phylogeny based on this gene indicated that *An. sawadwongporni* is more similar to *An. maculatus*, while *An. pseudowillmori* is more distantly related to these two species [17]. These results were consistent with the analysis of other molecular markers in previous studies including ITS2 of rDNA [11, 18] and combined ITS2 and D3 [16], which separated the *An. pseudowillmori* cluster from the *An. sawadwongporni* and *An. maculatus* clusters*.* Recently, these three species were identified by analyzing wing geometry which revealed consistent findings with DNA analysis, while modern morphometric findings were consistent with *cox*1 phylogenetic analysis and wing morphology [15]. Again, this finding based on the *cox*1 results is consistent with the current mosquito taxonomy, which placed *An. pseudowillmori* in the Maculatus group, and *An. sawadwongporni* and *An. maculatus* in the Sawadwongporni and Maculatus subgroups, respectively [9].

Our multiplex PCR results based on ITS2 were consistent with the *cox*1 gene findings which identified species according to their morphological identification. This confirms the accuracy of species identification of the Maculatus group by *cox*1 DNA barcodes. The *cox*1 sequences of the three potential malaria vectors in Thailand submitted to the GenBank database could be used as reference *cox*1 sequences in the understudied Maculatus group and for future taxonomic studies of *Anopheles* vectors.

## Conclusions

This study provided an update on the seasonal distribution of *An. maculatus* complex in malaria-endemic western Thailand. Both molecular techniques *cox*1 DNA barcoding and species-specific multiplex PCR based on the ITS2 region accurately identified three member species of the Maculatus group in Thailand. Successful species identification of malaria vectors affects the correct planning and implementation of control measures for *Anopheles* mosquitoes, required for integrated malaria control.

## Supplementary information

**Additional file 1: Table S1.** The information of 29 mosquito samples using for analysis; GenBank accession number sample codes, villages, and coordinates.

**Additional file 2: Table S2.** GenBank accession numbers of mosquitoes used for phylogenetic tree construction.

## Data Availability

The datasets used and/or analyzed during the current study are available from the corresponding author upon request.

## Abbreviations

BOLD: The Barcode of Life Database
CDC light trap: Centers for Disease Control and Prevention miniature light trap
CS: Circumsporozoite
*cox*1: Cytochrome *c* oxidase subunit 1
*cox*2: Cytochrome *c* oxidase subunit 2
DNA: Ribosomal deoxyribonucleic acid
ELISA: Enzyme-linked immunosorbent assay
ITS2: Internal transcribed spacer 2
K2P: Kimura 2-parameter model
KN: Komonae village
NB: Nong Bua village
PCR: Polymerase chain reaction
PV210: *Plasmodium vivax* 210
PV247: *Plasmodium vivax* 247
*s.l.*: *sensu lato*
SO: Suan Oi village
TO: Tala Oka village
