# Supplementary material for: Seasonal dynamics and molecular differentiation of three natural Anopheles species (Diptera: Culicidae) of the Maculatus group (Neocellia series) in malaria hotspot villages of Thailand
Source: Parasit Vectors. 2020 Nov 11;13:574. doi: 10.1186/s13071-020-04452-0 (PMC7659066; doi:10.1186/s13071-020-04452-0)
Supplement: Supplementary file 1 — Additional file 1: Table S1. The information of 29 mosquito samples using for analysis; GenBank accession number sample codes, villages, and coordinates. [file 13071_2020_4452_MOESM1_ESM.docx]

**Additional file 1: Table S1.** The information of 29 mosquito samples using for analysis; GenBank accession number sample codes, villages, and coordinates.

| **Mosquito species** | **GenBank accession no.** | **Sample code** | **Villages** | **GPS coordinates** |
| --- | --- | --- | --- | --- |
| *Anopheles maculatus* |  |  |  |  |
|  | 1. MK579204 | CODE 13825 | Komonae | 17°31'57.0"N 97°56'59.4"E |
|  | 2. MK579205 | CODE 4020 | Komonae | 17°31'55.2"N 97°56'59.9"E |
|  | 3. MK579206 | CODE 6546 | Komonae | 17°31'56.4"N 97°56'59.6"E |
|  | 4. MK579207 | CODE 6766 | Komonae | 17°31'55.2"N 97°56'59.3"E |
|  | 5. MK579208 | CODE 7986 | Komonae | 17°31'55.1"N 97°57'00.4"E |
|  | 6. MK579209 | CODE 8241 | Komonae | 17°31'57.0"N 97°56'59.4"E |
|  | 7. MK579210 | CODE 10389 | Komonae | 17°31'57.7"N 97°56'59.0"E |
|  | 8. MK579211 | CODE 10506 | Tala Oka | 17°19'23.3"N 98°07'00.6"E |
|  | 9. MK579212 | CODE 11650 | Komonae | 17°31'56.4"N 97°56'59.6"E |
| *Anopheles sawadwongporni* |  |  |  |  |
|  | 1. MK579213 | CODE 3733 | Noung Bua | 17°20'27.4"N 98°06'21.9"E |
|  | 2. MK579214 | CODE 4206 | Komonae | 17°31'57.4"N 97°56'57.5"E |
|  | 3. MK579215 | CODE 4609 | Suan Oi | 17°33'34.6"N 97°55'13.3"E |
|  | 4. MK579216 | CODE 6653 | Komonae | 17°31'57.7"N 97°56'59.0"E |
|  | 5. MK579217 | CODE 6654 | Komonae | 17°31'57.7"N 97°56'59.0"E |
|  | 6. MK579218 | CODE 6655 | Komonae | 17°31'57.7"N 97°56'59.0"E |
|  | 7. MK579219 | CODE 6790 | Komonae | 17°31'55.2"N 97°56'59.9"E |
|  | 8. MK579220 | CODE 10362 | Komonae | 17°31'55.7"N 97°56'56.4"E |
|  | 9. MK579221 | CODE 12778 | Noung Bua | 17°20'37.3"N 98°06'20.9"E |
| *Anopheles pseudowillmori* |  |  |  |  |
|  | 1. MK579222 | CODE 7706 | Tala Oka | 17°19'27.4"N 98°07'09.7"E |
|  | 2. MK579223 | CODE 10385 | Komonae | 17°31'57.7"N 97°56'59.0"E |
|  | 3. MK579224 | CODE 10575 | Tala Oka | 17°19'24.4"N 98°07'01.8"E |
|  | 4. MK579225 | CODE 10925 | Tala Oka | 17°19'26.2"N 98°07'12.0"E |
|  | 5. MK579226 | CODE 12715 | Noung Bua | 17°20'22.2"N 98°06'31.3"E |
|  | 6. MK579227 | CODE 12909 | Noung Bua | 17°20'23.6"N 98°06'22.1"E |
|  | 7. MK579228 | CODE 13767 | Komonae | 17°31'55.5"N 97°57'00.0"E |
|  | 8. MK579229 | CODE 14131 | Noung Bua | 17°20'26.6"N 98°06'21.9"E |
|  | 9. MK579230 | CODE 14217 | Noung Bua | 17°20'28.8"N 98°06'21.7"E |
|  | 10. MK579231 | CODE 14395 | Noung Bua | 17°20'37.3"N 98°06'20.9"E |
|  | 11. MK579232 | CODE 14783 | Tala Oka | 17°19'24.1"N 98°06'57.7"E |
