# Supplementary material for: Seasonal dynamics and molecular differentiation of three natural Anopheles species (Diptera: Culicidae) of the Maculatus group (Neocellia series) in malaria hotspot villages of Thailand
Source: Parasit Vectors. 2020 Nov 11;13:574. doi: 10.1186/s13071-020-04452-0 (PMC7659066; doi:10.1186/s13071-020-04452-0)
Supplement: Supplementary file 2 — Additional file 2: Table S2. GenBank accession numbers of mosquitoes used for phylogenetic tree construction. [file 13071_2020_4452_MOESM2_ESM.docx]

**Additional file 2: Table S2.** GenBank accession numbers of mosquitoes used for phylogenetic tree construction.

| **Species** | **Geographical location** | **GenBank accession no.** |
| --- | --- | --- |
| *An. sawadwongporni* | Tak, Thailand (SAW1–9) | MK579213-MK579221 |
| *An. maculatus* | Tak, Thailand (MAC1–9) | MK579204-MK579212 |
| *An. pseudowillmori* | Tak, Thailand (PSE1–11) | MK579222-MK579232 |
| *An. sawadwongporni* | Middle Hainan, China | JQ728407.1 |
| *An. sawadwongporni* | Middle Hainan, China | JQ728408.1 |
| *An. maculatus* | Mizoram, India | JN596972.1 |
| *An. maculatus* | Southern Yunnan, China | JQ728164.1 |
| *An. pseudowillmori* | Southern Yunnan, China | JQ728241.1 |
| *An. dravidicus* | Punjab, Pakistan | KF406679.1 |
| *Ae. aegypti* | Thailand | KP843391.1 |
| *Ae. albopictus* | Thailand | KP843399.1 |
| *Cx. quinquefasciatus* | Thailand | HQ398883.1 |
| *Ma. bonneae* | Thailand | HQ398879.1 |
